# Lung adenocarcinoma in a patient with a cis *EGFR* L858R‐K860I doublet mutation identified using NGS‐based profiling test: Negative diagnosis on initial companion test and successful treatment with osimertinib

**DOI:** 10.1111/1759-7714.13694

**Published:** 2020-10-09

**Authors:** Hiroto Onozawa, Haruhiro Saito, Kuniko Sunami, Takashi Kubo, Noboru Yamamoto, Rika Kasajima, Takashi Ohtsu, Yukihiko Hiroshima, Heiwa Kanamori, Tomoyuki Yokose, Yohei Miyagi

**Affiliations:** ^1^ Department of Thoracic Oncology Kanagawa Cancer Center Yokohama Japan; ^2^ Center for Cancer Genome Medicine Kanagawa Cancer Center Yokohama Japan; ^3^ Department of Laboratory Medicine National Cancer Center Hospital Tokyo Japan; ^4^ Department of Experimental Therapeutics National Cancer Center Hospital Tokyo Japan; ^5^ Cancer Treatment Division Kanagawa Cancer Center Research Institute Yokohama Japan; ^6^ Department of Pathology Kanagawa Cancer Center Yokohama Japan; ^7^ Molecular Pathology and Genetics Division Kanagawa Cancer Center Research Institute Yokohama Japan

**Keywords:** Doublet mutation, *EGFR*, K860I, L858R, non‐small cell lung cancer (NSCLC)

## Abstract

Tyrosine kinase inhibitors are used as first‐line treatment for non‐small cell lung cancer (NSCLC) patients harboring driver mutations in *EGFR*, *ALK*, *ROS1*, and *BRAF*. Currently, standard molecular testing approaches help identify single genes for such targetable driver mutations in NSCLC; however, next‐generation sequencing (NGS)‐based genetic profiling provides a more comprehensive approach and is hence strongly recommended. This case study aimed to highlight the benefits of NGS‐based tests for the diagnosis of complex *EGFR* L858R mutations. A patient was diagnosed with stage IVB NSCLC using a government‐approved in vitro diagnostic test and was noted to have a high programmed death‐ligand 1 tumor proportion score. This patient was treated with pembrolizumab monotherapy followed by cisplatin and pemetrexed owing to the lack of actionable driver gene mutations, including *EGFR* mutations. After treatment failure, a sample harvested from the same transbronchial lung biopsy specimen (formalin‐fixed and paraffin‐embedded) used for the initial *EGFR* test was subjected to NGS‐based broad genetic profiling. The NGS‐based test identified an *EGFR* L858R‐K860I cis doublet mutation; however, neither of these mutations was identified upon initial molecular testing. The patient was then successfully treated with a third‐generation EGFR‐tyrosine kinase inhibitor, osimertinib. In this study, we delved deeper into the realm of L858R and K860I mutations in NSCLC and discuss the potential causes underlying our initial negative diagnosis. Furthermore, this study highlighted the additional benefits of replacing typical molecular tests with NGS‐based broad profiling approaches.

**Key points:**

**Significant findings of the study:**

- The *EGFR* L858R‐K860I cis doublet mutation was not detected by a PCR‐based *EGFR* test.
- A next generation sequencing (NGS)‐based test was able to identify the L858R‐K860I cis doublet mutation.

**What this study adds:**

- Osimertinib was effective in an NSCLC patient with *EGFR* L858R and K860I mutations.

## Graphical abstract

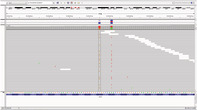

## Introduction

Thomas *et al*. first reported that non‐small cell lung cancers (NSCLCs) harboring *EGFR* mutations present a specific clinical response to tyrosine kinase inhibitors (TKIs).^1^ Since then, TKIs are used as the first‐line treatment for patients with NSCLC with driver mutations in *EGFR*, *ALK*, *ROS1*, and *BRAF*. Currently, government‐approved in vitro diagnosis tests (IVDs) are utilized to detect individual targetable driver gene mutations in NSCLC. However, next‐generation sequencing (NGS)‐based genetic profiling tests offer a broader scope and can identify numerous targetable genes simultaneously; thus, their use for cancer screening is gaining interest and is now strongly recommended.

## Case report

**Figure 1:**
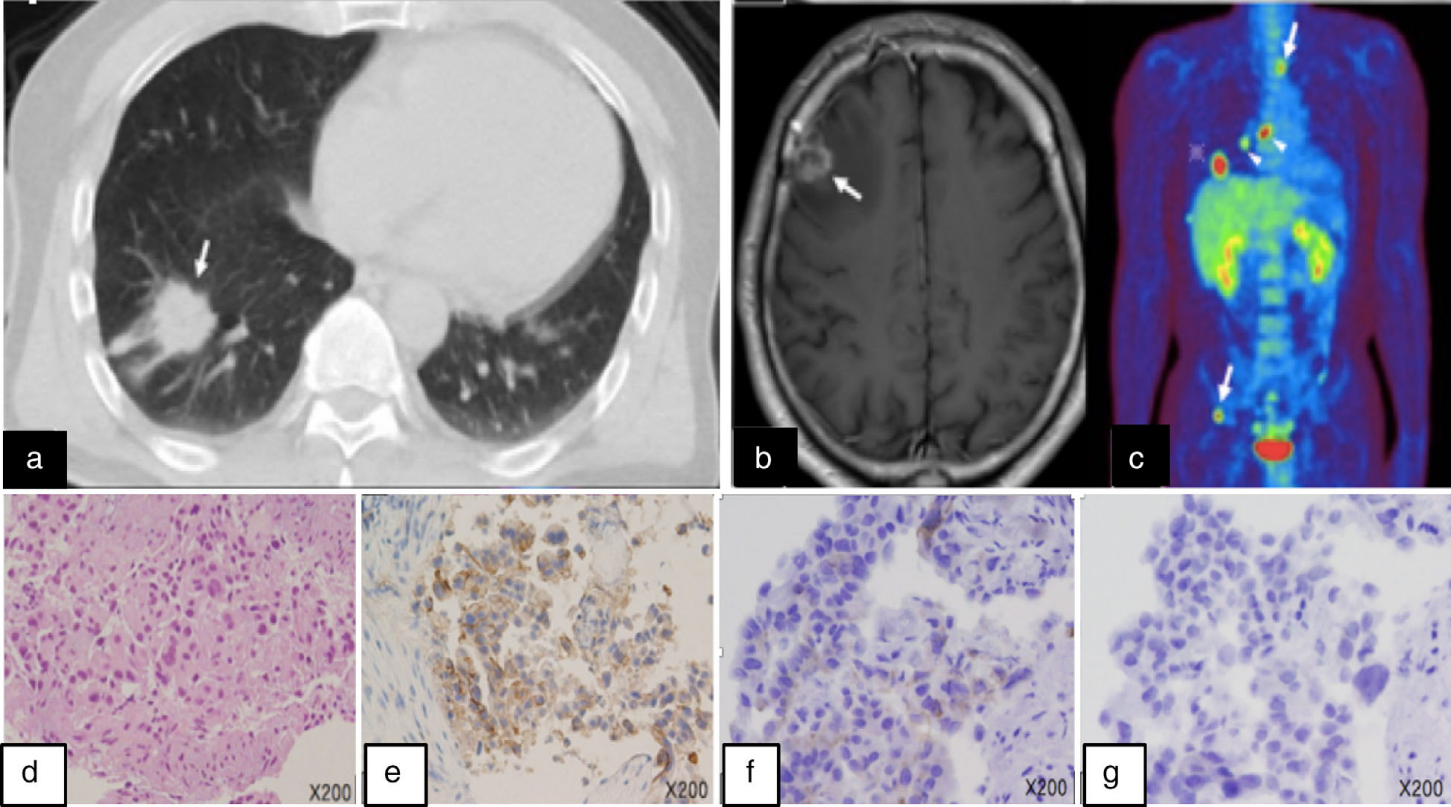
Imaging tests and histopathological findings of the transbronchial lung biopsy (TBLB) specimen. (**a**) Lung windows of axial chest CT showed a mass lesion measuring 6.4 cm × 2.9 cm × 2.6 cm with pleural indentation in the right S9. MRI (**b**) and positron emission tomography/CT (**c**) revealed brain and multiple lymph node and bone metastases (arrowheads). Hematoxylin‐eosin staining of the TBLB specimen demonstrated adenocarcinoma‐like histology with round malignant glands and a papillary growth (**d**); IHC revealed that PD‐L1 TPS was 70% (22C3 pharmDx Dako: Agilent Pathology Solutions, Santa Clara, CA, USA) (**e**). IHC analysis of the TBLB specimen with the EGFR L858R‐specific antibody (**f**), but not the exon 19 E746‐A750 deletion mutant‐specific antibody (**g**) (clone 43B2 and 6B6, respectively, rabbit monoclonal: Cell Signaling Technology, Danvers, MA, USA), demonstrated positive signals on the tumor cell membrane.

**Figure 2:**
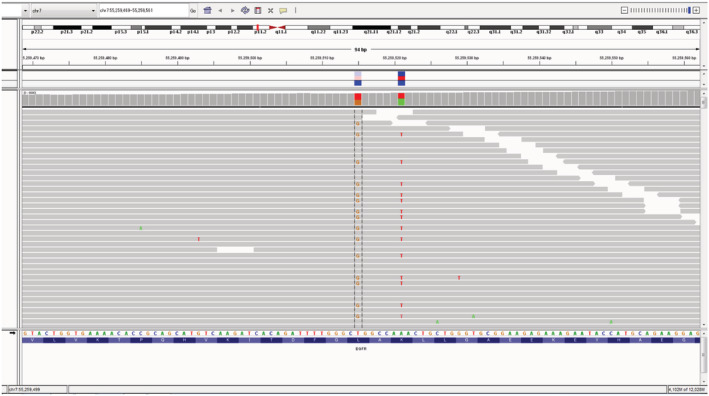
The integrative genomics viewer (IGV) image of *EGFR* nucleotide sequencing reads for the patient obtained by the NCC Oncopanel test. The nucleotide sequence and coding amino acids of wild‐type *EGFR* are shown at the bottom. Gray horizontal bars represent each sequence read, and clearly demonstrated that the c.2573 T > G (L858R) and c.2579 C > T (K860I) mutations were on the same allele.

**Figure 3:**
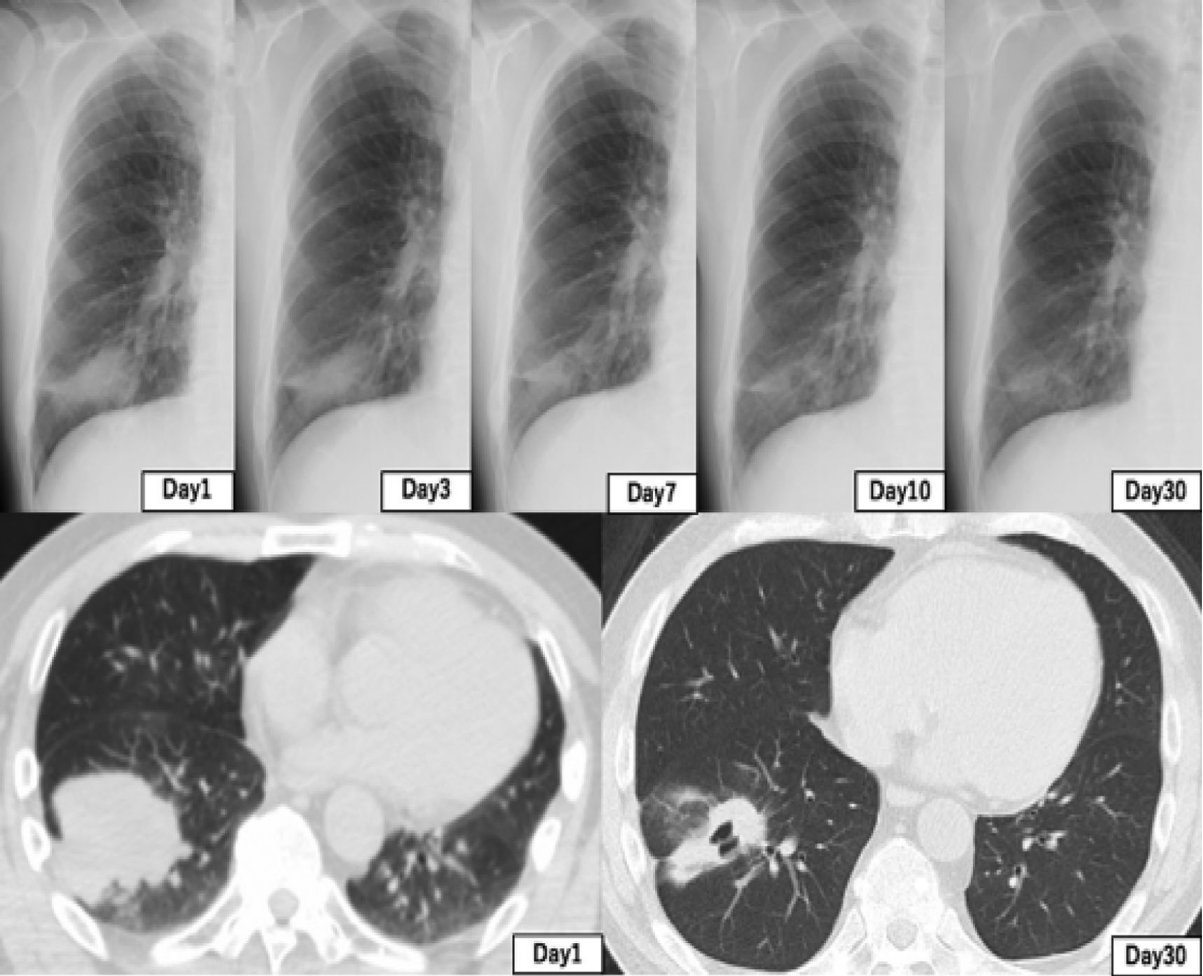
Computed tomography (CT) and chest X‐ray images showing response dynamics of the regrown primary tumor during osimertinib treatment. A decrease in tumor burden after one month was observed compared with that on day 1. The response was noticed within a few days of starting the treatment by chest X‐ray imaging.

A 56‐year‐old man with a past history of smoking (two cigarettes a day for 10 years) was referred to Kanagawa Cancer Center with an abnormal shadow on chest X‐ray. Computed tomography (CT) imaging revealed a 60 mm tumor in the right lower lobe (Fig 1a). Thereafter, a transbronchial lung biopsy (TBLB) was performed and the specimens were histologically examined; the patient was diagnosed with stage IVB (cT3N2M1) adenocarcinoma in accordance with the TNM classification of the Union of International Cancer Control (UICC), eighth edition. Moreover, precise imaging analyses revealed multiple lymph node metastases along with distant bone and brain metastases (Fig 1b,c). Formalin‐fixed and paraffin‐embedded (FFPE) TBLB tissue samples were then subjected to routine IVDs to detect *EGFR*, *AL*K, *ROS1*, and *BRAF* mutations, all of which were absent. Furthermore, The PD‐L1 TPS of this patient was 70% (Fig 1e). Thus, pembrolizumab monotherapy was initiated using our standard protocol. The patient underwent six treatment cycles of pembrolizumab, with three‐week intervals between consecutive cycles. However, this treatment regimen only managed to stabilize the disease; recurrence was evident after the sixth cycle, as both primary and metastatic tumors regrew. Thereafter, a second‐line combinatorial treatment regimen comprising two cycles of cisplatin and pemetrexed was initiated. Notably, chest CT imaging revealed a reduction in tumor size, which was accompanied by a reduction in carcinoembryonic antigen (CEA) serum levels. However, the patient experienced frequent watery diarrhea after the two cycles, which was diagnosed as delayed grade 3 colitis, an immune‐related adverse event (irA/E) caused by the preceding pembrolizumab treatment. Thus, the patient was administered 40 mg (0.5 mg/kg/day) of prednisolone sodium succinate and the irA/E was resolved. Nonetheless, spinal magnetic resonance imaging (MRI) and chest CT scans indicated the progression of both primary and secondary (metastasized) tumors. Thereafter, the patient was enrolled in NCCH1616 (UMIN000032166), a clinical study based on the Japanese advanced medical treatment system conducted at the National Cancer Center (NCC) Japan; the NCC Oncopanel test^1^ was performed using another FFPE sample prepared from the same TBLB tissue block used for the previous *EGFR* molecular test. The NCC Oncopanel test identified an *EGFR* L858R‐K860I doublet mutation in the cis configuration, ie, the two mutations were on the same allele (Table 1, Fig 2). Immunohistochemical (IHC) examination of the TBLB specimen with an EGFR L858R mutant‐targeted antibody (clone 43B2, rabbit monoclonal, Cell Signaling Technology, Danvers, MA, USA) revealed membrane expression of this mutant protein (Fig 1f,g). Accordingly, the patient was treated with osimertinib, a third‐generation TKI, which was effective and led to partial remission within one month. Interestingly, the effects were noticeable by chest X‐ray within only a few days of treatment initiation (Fig 3). Moreover, the patient tolerated the conventional doses of osimertinib, without any major side effects, for 6 months, after which a partial response was confirmed.

**Table 1:** Epidermal growth factor receptor (*EGFR*) mutations identified via next‐generation sequencing (NGS)‐based genomic profiling test

	c.2572 ‐ c.2580			Reads (frequency)	Alterations
nt. seq1	CTG	GCC	AAA	2404 (48.3%)	Wild‐type
amino acid	L	A	K	‐	Wild‐type
nt. seq2	CGG	GCC	ATA	1966 (39.5%)	c.2573 T > G, c.2579 A > T
amino acid	R	A	I	‐	L858R, K860I

nt. seq, nucleotide sequence.

## Discussion

Initially, the cobas *EGFR* mutation test v2 (Roche Molecular Systems, Pleasanton, CA, USA) was considered to screen the TBLB patient specimen.^2^ Although this test is designed to detect multiple *EGFR* mutations including two different L858R mutations, c.2573 T > G and c.2573_2574 TG > GT, it failed to identify the L858R mutation in our samples. However, K860I is a rare mutation that is not covered by the cobas test. Notably, screening another sample from the same TBLB tissue block using the NCC Oncopanel system identified L858R and K860I mutations on the same allele. The NCC Oncopanel is a hybridization capture and NGS‐based genomic profiling test that examines the entire coding regions of 114 cancer‐related genes along with 12 oncogene rearrangements. Evidently, the nucleotide alteration of the identified L858R mutation was c.2573 T > G, which should have been detected through the cobas test. Cobas is a multiplex allele‐specific PCR‐based test; considering the close proximity of the L858R and the K860I mutation sites, primers designed to detect the c.2573 T > G mutation might overlap the neighboring c.2579 A > T. One could speculate that the PCR primers were somehow disturbed by the adjacent c.2579 A > T K860I mutation, leading to a false‐negative diagnosis. We urge more studies to confirm our hypothesis and further investigate this phenomenon.

We compared our findings with data from the Catalogue Of Somatic Mutations In Cancer (COSMIC) database (v90). Notably, we noted eight other lung cancer cases reported to have *EGFR* L858R‐K860I doublet mutations, which we summarized in Table 2. These eight cases included six adenocarcinomas, one squamous cell carcinoma, and one adenosquamous carcinoma among these cases, four cases presented a doublet mutation in the cis configuration.^3^, ^4^, ^5^, ^6^, ^7^, ^8^, ^9^ Moreover, all of these cases were identified by NGS‐based methods, except for one in which a PCR‐based method was applied.^8^ The frequency of the confirmed L858R‐K860I doublet cis mutations in all *EGFR* mutations reportedly ranges 0–4%.^4^, ^7^, ^8^, ^10^ To our knowledge, the K860I mutation has never been reported alone, as it usually occurs as a compound mutation with L858R in cis configuration, except that it occurred as a doublet mutation with L861Q.^5^

**Table 2:** Reported *EGFR* L858R‐K860I double mutations in lung cancer

h. type	specimen	method	config.	initial TKI	res.	ref
ad	primary	NGS	cis	osimertinib	PR	this case
ad	pleural effusion	NGS	cis	erlotinib	PR	^3^
ad	ascites	NGS	cis	na	response^a^	^4^
ad	primary	PCR	ne	gefitinib	PR	^5^
ad	primary	PCR	ne	‐	‐	^6^
adsc	primary	PCR/NGS	cis	‐	‐	^7^
ad	primary	PCR^b^	cis	‐	‐	^8^
ad	metastasis	NGS	na	na	na	^9^
sc	primary	NGS	na	na	na	^9^

h. type, histological type; config., configuration; res., response; ad, adenocarcinoma; adsc, adenosquamous carcinoma; sc, squamous carcinoma; PCR, direct Sanger sequencing of PCR products; ne, not examined; na, not available in the literature; PR, partial response.

^a^ response in the literature but details unknown,

^b^ cis configuration was confirmed by cloning the PCR products.

Interestingly, two cases of L858R‐K860I doublet mutations were noted when re‐evaluating samples in studies through NGS‐based methods to investigate mechanisms underlying secondary resistance among patients responding to initial EGFR‐TKI treatments.^3^, ^4^ Although the configuration of this mutation was not stated, one doublet mutation case partially responded to gefitinib treatment.^5^ Furthermore, this study reports positive IHC reactivity to a mutated L858R‐specific antibody, suggesting that *EGFR* with a L858R‐K860I doublet mutation shares a similar activated conformation to *EGFR* with the L858R singlet mutation. Overall, patients harboring the L858R‐K860I doublet mutation may exhibit a response to EGFR‐TKI similar to that of patients with a singlet L858R mutation. An in vitro study evaluating the *EGFR* K860I singlet mutation introduced in Ba/F3 cells reported that the mutation was sensitive to osimertinib and partially sensitive to gefitinib and erlotinib.^10^

In this case study, an NSCLC patient harbored no *EGFR* mutation on the cobas PCR‐based test. However, after a poor response to a general chemotherapeutic drug regimen, an NGS‐based broad genetic profiling test was performed and led to the identification of a cis doublet *EGFR* mutation (L858R‐K860I). Thus, the third‐generation EGFR‐tyrosine kinase inhibitor osimertinib was selected to treat this patient; this treatment method was successful and led to partial remission within one month. Complex *EGFR* mutations in lung cancer are generally noted through NGS‐based molecular tests and Kohsaka *et al*. recently reported that almost one‐fifth of NSCLC cases with the *EGFR* L858R mutation (38/195 cases) actually harbor comutations with other parts of the *EGFR* gene.^10^ Although NGS‐based molecular profiling tests certainly benefited the present patient, NGS‐based tests must be considered initial molecular tests for NSCLC patients from the viewpoint of economic status and sensitivity. The unit cost of NGS‐based tests is markedly higher than that of individual single‐gene tests and actually the cost of the NCC Oncopanel is >20‐fold that of the Cobas test in Japan at present. For sensitivity issues, although the Cobas test failed to diagnose L858R‐K860I doublet mutations, it is generally more sensitive than NGS‐based tests for targeted *EGFR* mutations including T790M (data from the Roche Molecular Systems, Inc., provided in the attachment of the Cobas *EGFR* mutation Test v2). However, when used as an initial molecular test, NGS‐based molecular tests may save time for patients in identifying driver gene mutations targetable with already available drugs, including genes not known to be commonly involved in the pathogenesis of NSCLCs. NGS‐based molecular tests have recently been approved by governments of various countries, including IVDs, including NCC Oncopanel in Japan. Although NGS‐based molecular tests have to be performed considering numerous aspects and the status of the current NSCLC patients, decreasing the cost and improving the sensitivity may facilitate the application of these tests as an initial molecular test for at least NSCLCs.

## Disclosure

The authors declare that there are no conflicts of interest.
